# Rapid PCR detection of group a streptococcus from flocked throat swabs: A retrospective clinical study

**DOI:** 10.1186/1476-0711-10-33

**Published:** 2011-09-02

**Authors:** Robert Slinger, David Goldfarb, Derek Rajakumar, Ioana Moldovan, Nicholas Barrowman, Ronald Tam, Francis Chan

**Affiliations:** 1 Department of Laboratory Medicine, Children's Hospital of Eastern Ontario, University of Ottawa, Ottawa, ON, Canada; 2 Department of Pediatrics, University of Botswana, Gaborone, Botswana; 3 Research Institute, Children's Hospital of Eastern Ontario, University of Ottawa, Ottawa, ON, Canada

**Keywords:** PCR, rapid, internally-controlled, LCGreen, Group A Streptococcus, pharyngitis, flocked swab

## Abstract

**Background:**

Rapid diagnosis of GAS pharyngitis may improve patient care by ensuring that patients with GAS pharyngitis are treated quickly and also avoiding unnecessary use of antibiotics in those without GAS infection. Very few molecular methods for detection of GAS in clinical throat swab specimens have been described.

**Methods:**

We performed a study of a laboratory-developed internally-controlled rapid Group A streptococcus (GAS) PCR assay using flocked swab throat specimens. We compared the GAS PCR assay to GAS culture results using a collection of archived throat swab samples obtained during a study comparing the performance of conventional and flocked throat swabs.

**Results:**

The sensitivity of the GAS PCR assay as compared to the reference standard was 96.0% (95% CI 90.1% to 98.4%), specificity 98.6% (95% CI 95.8% to 99.5%), positive predictive value (PPV) 96.9% (95% CI 91.4% to 99.0%) and negative predictive value (NPV) of 98.1% (95% CI 95.2% to 99.2%). For conventional swab cultures, sensitivity was 96.0% (95% CI 90.1% to 98.4%), specificity 100% (95% CI 98.2% to 100%), PPV 100%, (95% CI 96.1% to 100%) and NPV 98.1% (95% CI 95.2% to 99.3%)

**Conclusions:**

In this retrospective study, the GAS PCR assay appeared to perform as well as conventional throat swab culture, the current standard of practice. Since the GAS PCR assay, including DNA extraction, can be performed in approximately 1 hour, prospective studies of this assay are warranted to evaluate the clinical impact of the assay on management of patients with pharyngitis.

## Background

Rapid diagnosis of *Streptococcus pyogenes*, commonly referred to as group A streptococcus (GAS), from throat specimens may be useful for patient management, both ensuring that patients with GAS pharyngitis are treated quickly and also avoiding unnecessary use of antibiotics in those without GAS infection. Surprisingly, there have been very few reports of nucleic acid (NA) amplification assays for rapid GAS detection from throat swabs [1,2]. The lack of published reports in this area may reflect concerns about the costs and complexity of NA amplification methods relative to culture and antigen detection methods.

We therefore designed an internally-controlled, rapid and relatively inexpensive PCR assay for GAS pharyngitis, and retrospectively evaluated this assay using archived throat swab specimens. The assay made use of recent innovations in specimen collection and nucleic acid amplification by using the new flocked swabs for specimen collection and by using a newer intercalating dye, LC Green, for amplicon detection [3,4].

## Methods

### PCR primers and internal amplification control design

We chose the *dnaseB *gene as our target since this gene appears to be both present in all *S. pyogenes *and also unique to this organism [5]. We designed a PCR primer pair to amplify a *dnaseB *gene sequence using the Primer3 program [6]. In order to determine if PCR inhibition was present in any specimens, we designed a competitive internal amplification control (IAC) oligonucleotide that would be amplified by the GAS primers selected, but have a lower melting temperature (*T*_m_)_. _The primers and the IAC sequences are shown in Table 1.

**Table 1:** Primers and internal amplification control (IAC) oligonucleotide sequences (5'- 3' direction) for group A streptococcus (GAS) *dnaseB *assay

*dnaseB *forward primer	TGA TTC CAA GAG CTG TCG TG
*dnaseB *reverse primer	TGG TGT AGC CAT TAG CTG TGT T
IAC	TGATTCCAAGAGCTGTCGTGatcaatataacaaacacttgcatatatatact tacgaaactaataactaaataatcaatataaatACACAGCTAATGGCTACACCA

### PCR protocol

The primers and the internal control oligonucleotide were synthesized by Integrated DNA Technologies, Inc (Coralville, ID). PCR reactions were performed in 10 μL volume, including 4 μL of Light Scanner master mix containing the LCGreen Plus + dye (Idaho Technology Inc., Salt Lake City, UT), a 0.5 μmol/L concentration of each primer, a 1 picomolar concentration of internal control oligonucleotide and 1 μL of specimen DNA.

The thermocycler (RapidCycler II, Idaho Technology Inc.) protocol consisted of 40 cycles of 95°C denaturation, 60°C annealing, 72°C extension with a 10 second hold at each temperature and a programmed transition rate of 9.9°C per second, with a total run time of 35 minutes.

After PCR, samples were transferred to the high-resolution melting instrument (HR-1). A rate of 0.3°C per second was used when measuring the fluorescence change of the sample in a temperature range of 60°C to 90°C. The derivative of the measured fluorescence plotted against temperature was displayed using the HR-1 software and the peak of these curves was measured to obtain the amplicon *T*_m_.

### Specificity panel

Prior to clinical specimen testing, we verified primer specificity *in silica *using the BLAST tool, and *in vitro *against a panel of DNA extracts from a collection of reference bacterial strains. These included *Streptococcus dysgalactiae *subsp. *equisimilis *(large-colony Lancefield antigen Group C and G positive beta-hemolytic streptococci), *S. agalactiae *(Group B streptococcus), *S. intermedius, S. constellatus, S. anginosus, S. pneumoniae *ATCC 49619, and *Streptococcus salivarius *ATCC 13419 *Escherichia coli *American type culture collection (ATCC) 25922, *Haemophilus influenzae *ATCC 49766, *H. influenzae *ATCC 49247, *Haemophilus parainfluenzae *ATCC 7901, *K. pneumoniae *ATCC 700603, *Moraxella catarrhalis *ATCC 25238, *Staphylococcus aureus *ATCC 29247, *Neisseria gonorrhoeae *ATCC 49226, *Neisseria lactamica *ATCC 23970, *Pseudomonas aeruginosa *ATCC 27853, *Enterococcus faecalis *ATCC 29212.

### GAS PCR retrospective clinical specimen study

We used specimens for the PCR evaluation that had been previously collected as part of a study comparing flocked swabs to conventional swabs for detection of GAS by culture [3]. Physicians obtained throat specimens from patients seen in the Children's Hospital of Eastern Ontario (CHEO) Emergency Department, using dual swabs with flocked and conventional swab attached to the same base. The initial study was conducted from September to December 2007. Research Ethics Board approval was obtained and consent was required from each participant.

After samples were collected, the flocked swab was removed from the dual swab applicator base and placed into a transport tube containing 1 ml Liquid Stuarts transport media. The conventional swab was placed in a traditional throat swab transport tube. Both tubes were then sent to the on-site bacteriology laboratory.

The conventional swab was used to directly streak a 5% blood agar (BA) plate. The transport tube containing the flocked swab was briefly vortexed. The flocked swab was then used to directly streak a second BA plate and 100 μL of the liquid media was streaked onto a third BA plate. Plates were incubated anaerobically for 24 hours prior to examination. Group A streptococci were identified by hemolysis, colony size, latex agglutination and bacitracin susceptibility. Five-hundred microliters of the liquid transport media was saved frozen at -80°C for future DNA extraction.

### GAS PCR study specimens

Three-hundred forty-four dual swabs were collected in the swab comparison study. Six swabs were excluded from analysis since they were improperly collected. Of the 338 liquid transport media specimens saved, 32 were consumed in preliminary comparisons of a variety of nucleic acid extraction methods. Three-hundred and six (90.5%) of the properly collected fluid specimens were thus available for testing with the GAS PCR assay.

### DNA extraction

The saved volume of flocked swab transport medium was thawed and then centrifuged for 3 minutes at 16000 × *g*. The supernatant was removed and 40 μL of PrepMan Ultra reagent (Applied Biosystems, Foster City, CA) was added to the pellet and vortexed for 30 seconds. This was then heated at 100°C for 10 minutes, and then centrifuged again for 3 minutes at 16000 × *g*. The supernatant was transferred to a new tube and saved at -80°C until testing.

### Repeatability and reproducibility

To assess repeatability, four PCR and culture positive and two negative clinical samples were run in triplicate twice in one day by the same operator using the same lot of master mix [7]. We used two specimens from which 1-10 CFU of GAS were grown in culture as "weak positives" to challenge the assay. To assess reproducibility, the same samples were then tested in triplicate one week later by a different technologist using a different lot of master mix [7].

### Main outcome measure and statistical analysis

The main outcome measures of the study were the performance characteristics (sensitivity and specificity, positive and negative predictive values) of a) the PCR assay and b) the conventional swab culture in comparison to the reference standard results. For statistical analysis, we used as the reference standard a positive culture for GAS from either or both of the two BA plates inoculated from the flocked swab specimen (with the transport liquid or directly with the flocked swab). Confidence intervals for performance characteristics were computed using the Wilson score method [8]. The differences between sensitivities were evaluated with the McNemar's test.

## Results

### GAS PCR retrospective clinical specimen study (Table 2)

The sensitivity of the GAS PCR assay as compared to the reference standard was 95/99 (96.0%, 95% CI 90.1% to 98.4%) with a specificity of 204/207 (98.6%, 95% CI 95.8% to 99.5%). The positive predictive value (PPV) was 95/98 (96.9%, 95% CI 91.4% to 99.0%) and the negative predictive value (NPV) 204/208 = (98.1%, 95% CI 95.2% to 99.2%). Regarding the four PCR-negative reference assay-positive specimens, cultures of the flocked swab liquid media, from which the DNA was extracted for PCR, grew GAS in very light quantities in 3 specimens and was GAS culture-negative from the fourth specimen.

**Table 2:** Group A streptococcus (GAS) *dnaseB *PCR and conventional swab culture results compared to the reference standard

		Flocked swab cultures*	
		**+**	**-**
		
Group A streptococcus *dnaseB *PCR assay	+	95	3
	-	4	204

Conventional swab culture	+	95	0
	-	4	207

*positive GAS culture on ≥ 1 of the 2 flocked swab blood agar plate cultures

For the conventional swab cultures, sensitivity was also 95/99 (96.0%, 95% CI 90.1% to 98.4%), with a specificity of 207/207 (100%, 95% CI 98.2% to 100%), PPV of 95/95 (100%, 95% CI 96.1% to 100%) and NPV of 207/211 (98.1%, 95% CI 95.2% to 99.3%)

**Figure 1:**
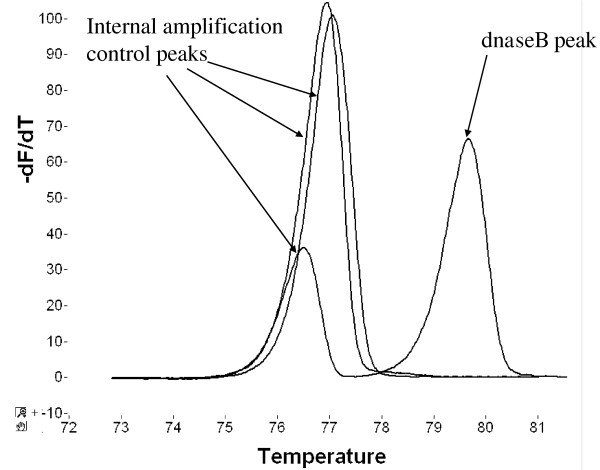
**Group A streptococcus (GAS) PCR derivative melting curves for three flocked throat swab specimens (one GAS PCR-positive and two GAS PCR-negative specimens)**. The GAS-PCR positive specimen shows both an internal amplification control (IAC) and *dnaseB *peak. Only IAC peaks are seen in the GAS-negative specimens, demonstrating that GAS was not detected and, in addition, that the PCR reaction was not inhibited.

The mean *T*_m _for the *dnaseB *peaks was 79.76°C (SD = 0.35°C). The mean *T*_m _for the IAC peaks was 76.89°C (SD = 0.36°C). Figure 1 illustrates examples of the derivative melt curves for one GAS PCR-positive and two GAS PCR-negative throat swab specimens.

### Indeterminate results

Melting peak graphs for ten specimens of 306 (3.3%, 95% CI 1.8% to 5.9%) were judged to be indeterminate and required repeat PCR testing. This group of ten included two specimens with neither IAC nor *dnaseB *peaks seen on the initial PCR run, and eight specimen results that showed peaks with heights between 5-15 units within the melting temperature range of the *dnaseB *amplicon. On repeat testing of these 10 specimens, all 10 gave results which could be readily interpreted. (Eight were found to be GAS PCR-negative and two were GAS PCR-positive).

Of note, since a competitive internal control design was used, the presence of either the IAC amplicon *or *the *dnaseB *amplicon indicated no PCR inhibition had occurred. Among the *dnaseB- *positive specimens, both *dnaseB *and IAC peaks were seen in 36/98 (36.7%) and the *dnaseB *peak alone in 62/98 (63.3%).

### Specificity panel and repeatability and reproducibility

The specificity panel study showed no amplicons that could lead to false positive GAS results. The 18 qualitative (positive or negative) PCR results were identical for the tests performed twice on the same day to assess repeatability as well as for those obtained when testing was performed one week later by a different operator with a different master mix lot to assess reproducibility.

## Discussion

The rapid internally-controlled GAS PCR assay described performed as well as the currently accepted standard method of conventional swab culture for GAS detection. PCR assay sensitivity of 96% was also similar to that seen in two prior prospective studies comparing PCR to culture GAS detection from throats swabs. In a study using dot blot and Southern Blot detection of PCR products from throat swabs, GAS detection sensitivity was also 96% [1], while the sensitivity of a fluorescent probe-based PCR detection method was 93% (82-98% CI) with a specificity of 98% (96-99% CI) [2].

Although a small number of specimens were reference assay- positive/PCR-negative, culture results from these specimens indicated that only specimens containing very low quantities of GAS may possibly be missed by PCR assay. As well, colonization of the pharynx with GAS is relatively common in pediatric patients, and recovery of GAS in low quantities by culture may not be clinically significant, as GAS may be a colonizer rather than the cause of pharyngitis in some cases. The high specificity of the GAS PCR assay ensures laboratories will only rarely encounter the dilemma of PCR-positive, culture-negative specimens.

The GAS PCR assay may be useful for clinical laboratories. It is relatively rapid, since it can be performed in approximately one hour, including the DNA extraction procedure, and as a closed-tube method, has a low risk of environmental cross-contamination. However, the assay is less suitable for medical office settings as compared to rapid antigen detection tests since more manual steps are required and since the turnaround time may be longer. The per reaction cost for the GAS LCGreen assay is low, at approximately $2.40 US, including DNA extraction and PCR reagents and consumables, but not including swab costs and personnel time.

The GAS PCR assay also demonstrates for the first time the clinical usefulness of flocked swabs in liquid transport media for bacterial molecular assays. Flocked swabs are created by attaching individual short fibers to an applicator, rather than winding long fibers around the applicator, as with conventional swabs. Due to this design, flocked swabs release a larger volume of specimen into the liquid transport media than conventional wound fiber swabs. This liquid media can then be used for nucleic acid extraction for molecular assays. Molecular testing from flocked swab clinical samples has previously been reported for detection of viral agents [9], but to our knowledge, this is the first report to demonstrate the successful use of PCR from flocked swab specimens for bacterial identification using true clinical specimens. (A study using serial dilutions of bacterial reference strains has shown improved sensitivity for nucleic acid amplification assays using flocked swabs [10].)

This assay also provides another example of the usefulness of LCGreen dye for amplicon detection and melting analysis. This dye has been used successfully for applications such as duplex and multiplex PCR where older SYBR Green dyes may be sub-optimal [4,5]. The SYBR Green dyes have been described, to have a tendency to bind preferentially to certain amplicons or due to inhibition of PCR by the dye itself [11]. In contrast, the LCGreen dye has been used successfully to detect and differentiated amplicons in duplex and multiplex PCR assays for the identification of infection agents, such as *Aspergillus *spp. and *Mycobacterium *spp., and for detection of antibiotic resistance genes [4,12,13]. LCGreen assay could be adapted successfully to any of several real-time thermocyclers compatible with this dye [14], so the GAS PCR we describe should be readily transferable to these instruments.

There are several limitations to our study. First, not all specimens collected in the original flocked swab study were available for testing with the PCR assay. However, the proportion positive for GAS by culture in the original study and in this study were similar, suggesting that this factor would not introduced bias into the PCR study. Secondly, samples were tested retrospectively after being saved frozen rather than being tested immediately after collection. We cannot exclude the possibility that freezing and thawing may have affected study results in some way.

A third limitation is that not all samples positive by the reference standard of positive flocked swab cultures were detected. The PCR sensitivity was, however, equal to that of a wound fiber conventional swab culture, which is currently the standard of practice in clinical microbiology laboratories.

## Conclusion

The GAS PCR assay appears to be an accurate and rapid molecular assay for GAS detection in children and adolescents with pharyngitis. Since the GAS PCR assay, including DNA extraction, can be performed in approximately 1 hour, prospective studies of this assay are warranted to evaluate the clinical impact of the assay on the management of patients with pharyngitis.

## Competing interests

The authors declare that they have no competing interests.

## Authors' contributions

RS drafted the primary manuscript. RS and DG designed the PCR assay. NB performed statistical analyses. DG, RT, FC contributed to study design and data collection and analysis. DR and IM participated in the performance of the PCR assay and data analysis. All authors contributed to the preparation of the manuscript. All authors read and approved the final manuscript.
